# Validation of Nepali Version of the University of Washington Quality of Life Questionnaire for Head and Neck Cancer Patients

**DOI:** 10.31729/jnma.9053

**Published:** 2025-06-30

**Authors:** Deepak Paudel, Anil Bikram Karki, Bibas Parajuli

**Affiliations:** 1 Department of ENT & HNS, BP Koirala Institute of Health Sciences, Dharan, Nepal; 2 Department of Surgical Oncology, Head and Neck Division, BP Koirala Memorial Cancer Hospital, Bharatpur, Nepal; 3 Butwal Provincial Hospital, Butwal, Nepal

**Keywords:** *head and neck cancer*, *quality of life*, *questionnaires*, *reliability*, *validity*

## Abstract

**Introduction:**

Quality of Life encompasses physical, functional, emotional, and social well-being, often assessed via self-reported questionnaires. The University of Washington Quality of Life instrument, designed for Head and Neck Cancer patients, is a validated, concise, and practical tool widely used in clinical settings. The objective of the study was to translate and assess the content and face validity of the Nepali version of the University of Washington Quality of Life Questionnaire version 4.1-N and evaluate its psychometric properties in head and neck cancer patients.

**Methods:**

A prospective study was conducted at BP Koirala Institute of Health Sciences and BP Memorial Cancer Hospital, enrolling 203 patients. The study was conducted in two phases. In the first phase, we translated the original questionnaire into Nepali and validated its face and content validity. In the second phase, we assessed its internal consistency, reliability, construct, and discriminant validity in Head and Neck Cancer patients.

**Results:**

The University of Washington Quality of Life Questionnaire version 4.1-N demonstrated strong internal consistency (Cronbach's α = 0.87), and excellent test-retest reliability and stability (r = 0.93). A strong correlation (r > 0.50) was observed between similar domains of the University of Washington Quality of Life Questionnaire version 4.1-N and EORTC QLQ-C30 and HN355. UWQOL composite scores demonstrated a strong correlation with all domains of global questions at admission, as well as after 3 and 6 months of treatment (p < 0.01).

**Conclusions:**

The Nepali version of University of Washington Quality of Life Questionnaire version 4.1-N is a valid and reliable instrument to assess the quality of life of head and neck cancer patients patients in Nepal.

## INTRODUCTION

Head and neck cancers (HNCs) are the ninth most common malignancy globally and account for 30% of all cancers in South Asia, with oral cancer being the most prevalent.^1, 2^ These cancers arise from regions critical for speech, swallowing, and breathing, and both the disease and its treatment significantly affect patients' quality of life (QoL).^3-5^ Health-related quality of life (HQL) has become an essential outcome measure in cancer care.^6,7^

QoL encompasses a multidimensional construct including physical, emotional, functional, and social well-being.^8^ While treatments may offer similar survival rates, their impact on QoL can differ. Management should aim not only for cure but also for preserving function and appearance. Pre-treatment discussions should prioritize post-treatment QoL outcomes.

The University of Washington Quality of Life (UWQOL) questionnaire is a brief, reliable, and validated tool for assessing QoL in HNC patients. ^9^ This study aimed to translate and validate the UWQOL version 4.1 into Nepali for use in Nepali-speaking populations.

## METHODS

This prospective study was conducted in two phases after the approval of the Institutional Review Board of BP Koirala Institute of Health Sciences (84/077/078-IRC) and the National Health Research Council (319/2021 MT). The study was conducted as per Helsinki's declaration. The duration of the study was from June 1, 2021, to May 31, 2024. In Phase 1, linguistic and cultural translation of the UWQOLv4.1 from English to Nepali was carried out. The translated version was prospectively validated in the second phase at BP Koirala Institute of Health Sciences, Dharan, and BP Memorial Cancer Hospital, Bharatpur, Nepal. Patients aged 18 years or older diagnosed with head and neck cancer who underwent primary surgical treatment were included in the study. Patients were excluded if they had recurrent disease, underwent revision surgeries, or declined to participate.

The UW-QOL v4.1 comprises 14 domains. Following the commonly accepted guideline of including 10 participants per item, the minimum required sample size was 140. To ensure sufficient statistical power, account for potential loss to follow-up, and allow for robust psychometric analyses, we planned to recruit a total of 200 participants for this study.

In phase one, the translation process began with a forward translation of the original English questionnaire into Nepali. Two Nepali native speakers did this with bicultural expertise in Nepal and the United States. Afterwards, a bilingual coordinator reviewed the cultural appropriateness of each version, and a consensus version was created. This version was then sent to two translators who performed a back translation from Nepali to English. The coordinator synthesized the two English translations into a final version of the questionnaire, which was compared with the original English version to ensure both linguistic accuracy and the preservation of nuanced meanings.

In phase two, five content experts, two head and neck surgeons, one medical oncologist, one radiation oncologist, and one oncology nurse, evaluated the translated questionnaire's face and content validity. Twenty eligible patients from outpatient clinics and wards established additional face validity. Patients reported that the Nepali version of UWQOLv4.1 (UWQOLv4.1- N) was easily understood and reflected most of their problems.

To obtain construct validity, it was hypothesised that the UWQOLv4.1-N would demonstrate correlations with similar domains of the European Organisation for Research and Treatment of Cancer Quality of Life Questionnaire-Head and Neck 35(EORTC QLQ- H&N35). The EORTC QLQ-H&N35 have been validated in multiple languages, including Nepali. Identifying significant correlations between these instruments and the UWQOLv4.1-N would support the UWQOLv4.1-N as a valid tool for assessing the quality of life in head and neck cancer patients, demonstrating robust construct validity. For discriminant validity, it was hypothesized that the UWQOLv4.1-N would effectively distinguish between patients at different stages of cancer. Additionally, it was anticipated that patients with varying cancer types and treatment modalities would exhibit distinct response patterns to individual items on the UWQOLv4.1-N. It was expected that these differences to be reflected in the UWQOLv4.1-N scores, thereby demonstrating the questionnaire's strong discriminant validity. Reliability was established by measuring both internal consistency (Cronbach's a) and test-retest reliability. Internal consistency is considered good if alpha approximates 0.70 but does not exceed 0.90, because values over 0.90 imply the presence of redundant items.^10^ Test-retest reliability and stability of the questionnaire were measured with the Spearman correlation.

Each item on the UW-QOLv4.1 is scored on a scale from 0 to 100, with higher scores indicating better quality of life. A composite score is calculated as the average of the 14 items, while three global questions are analysed separately. Similarly, items on the EORTC H&N35 module are scored from 0 to 100. On the functional scale, higher scores represent better quality of life, whereas on the symptom scale, higher scores indicate poorer quality of life.^8^

Demographic data (age, gender etc.), oncological data (site and stage of cancer), and treatment data (type of surgery and adjuvant Radiotherapy) of the patients were collected. A total of 237 patients initially enrolled in the study, 203 completed the 6-month follow-up and were included in the final analysis. Patients' quality of life was assessed using UWQOLv4.1-N at the initial evaluation, and again after 3 and 6 months of treatment. Additionally, patients were asked to complete the same questionnaire 10 days after the initial interview to assess test-retest reliability. This interval was chosen as it is generally sufficient to minimize recall of previous responses, while being short enough to prevent any clinically significant changes in the patient's condition during that period.

The UWQOLv4.1 (Nepali version) included 14 functional items related to head and neck cancer, each representing a specific domain. Each domain-specific item was scored on a scale from 0 to 100, where 0 indicated the worst quality of life and 100 the best.

Participants were also asked to select the three most important domains they experienced over the past seven days. Additionally, the UWQOLv4.1-N included four generic quality-of-life questions. These comprised three Likert-type items assessing health-related quality of life (HR-QOL) one month before the cancer diagnosis (Transitional HR-QOL), HR-QOL during the past seven days (Global HR-QOL), and overall quality of life during the past seven days (Global QOL).

Data were entered into Microsoft Excel 2019 (Microsoft, Redmond, WA, USA) and analyzed using IBM SPSS Statistics for Windows, version 24 (IBM Corp., Armonk, NY, USA). The principal investigator periodically reviewed the data to ensure completeness. Descriptive statistics were used to summarise the data, including frequencies, percentages, means and standard deviations. Since some scores were not normally distributed, non-parametric statistics methods were used. Internal consistency was evaluated using Cronbach's a. To assess convergent validity, spearman's correlation was used to examine the relationship between the UW-QOLv4.1-N and corresponding domains of the EORTC QLQ- C30 and Head and Neck 35 questionnaires. Discriminant validity was evaluated by comparing groups (based on cancer stage and treatment) using the Mann-Whitney U test. Due to the limited number of categories, the median was deemed an insensitive measure, and the mean was used to summarize domain scores.^11^

## RESULTS

The majority of participants were male, comprising 154 individuals (75.86%), resulting in a male-to-female ratio of 3.14:1. Most patients were diagnosed with carcinoma of the oral cavity (87.19%), while the remaining cases involved carcinoma of the larynx, hypopharynx, and lip. The mean age of the participants was 53.16 ± 8.76 years. A significant proportion of patients (83.25%) presented with advanced-stage disease, (Table 1).

**Table 1:** Demographic and clinical characteristics of patients (N = 203)

VARIABLE	n(%)	MEAN (SD)	RANGE (SD)
Age (years)		53.16 (8.76)	35-78
< 40	13(6.40)		
41-50	53(26.11)		
51-60	101(49.75)		
61-70	27(13.30)		
>70	9(4.43)		
**Gender**			
Male	154 (75.86)		
Female	49 (24.14)		
**T Stage**			
T1	13(6.40)
T2	21(10.34)		
T3	67(33.00)		
T4	102(50.24)		
**Site**			
Oral Cavity	177(87.19)		
Buccal Mucosa and alveolus	114(64.40)		
	63(35.59)		
Tongue	10(4.92)		
Larynx	9(4.43)		
Hypopharynx	7(3.45)		
Lip			
**Extent of Surgery**			
WLE+ND+PC	61(30.04)		
WLE+ND+PF	75(36.94 )		
WLE+ND+FF	67(33.010)		
**Radiotherapy**			
Yes	131(64.53)		
No	72(35.47)		

WLE = Wide Local Excision, ND = Neck dissection, PC = Primary Closure, FF = Free Flap

## INTERNAL CONSISTENCY OFTHE UW-QOLV4.1-N

Cronbach a for the UWQOLv4.1-N overall scale was 0.87. The items to total correlations of the UWQOLv4.1-N were all >0.30 and ranged from 0.32 to 0.69, (Table 2).

**Table 2:** Reliability Measures of Nepali Version of UWQOL, (N=203)

Item	Corrected Item-Total correlation	Cronbach's α if Item Deleted
Chronbach's α		0.87
Pain	.558	.857
Appearances	.341	.866
Activity	.603	.855
Recreation	.692	.849
Swallowing	.647	.852
Chewing	0.617	0.853
Speech	0.579	0.856
Shoulder	0.518	0.858
Taste	0.539	0.857
Saliva	0.329	0.872
Mood	0.588	0.854
Anxiety	0.527	0.858
Recreation	0.373	0.868
Fear of recurrence	0.608	0.853

## CONSTRUCT VALIDITY AND DISCRIMINANT VALIDITY

As there is no composite score calculated for EORTC H & N 35, a comparison between related domains of EORTC H&N 35 and UWQOL was made. Correlations were observed (r>0.50) between all similar domains, including pain, appearance, swallowing, chewing, speech, saliva, and shoulder items on the UWQOL(Table 3). The UWQOL composite scores correlated significantly with the global question on overall, HRQOL during the past 7 days and Overall QOL during the past 7 days at admission, after 3 months and 6 months of treatment (p<0.01) which further supports the construct validity (Table 4).

The UWQOLv4.1-N scores varied depending on the tumour stage and the type of treatment patients underwent (surgery alone or surgery followed by adjuvant radiotherapy). Patients with early-stage tumour showed significantly better scores across all quality of life (QoL) domains and composite scores (P < 0.01). Additionally, patients who received adjuvant radiotherapy after definitive surgery generally had lower QoL scores compared to those who underwent surgery alone. This difference was particularly significant in the domains of pain, appearance, and anxiety (Table 5).

**Table 3:** Correlation between Item-specific University of Washington Quality of Life Scores and EORTC QLQ C 30 HNQ35 subscale 3 months after surgery.

UWQOL subscale	EORTC subscales					
	Pain(p-value)	Appearance(p-value)	Saliva(p-value)	Chewing(p-value)	Swallowing (p-value)	Shoulder(p-value)
Pain	-0.77(<0.001)					
Appearance		-0.50(<0.01)				
Saliva			-0.56(<0.01)			
Chewing				-0.53(<0.01		
Swallowing					-0.62(<0.001)	
Shoulder						-0.64(<0.01)

Spearman correlation was used to correlate the related domains.

**Table 4:** Correlation between Composite score and global score at baseline, after 3 months and six months of treatment.

Composite Score	Global score		
	HRQOL Compared to a month before cancer(p-value)	HRQOL during the past 7 days (p-value)	Overall QOL during the past 7 days (p-value)
At admission	0.54(<0.01)	0.51(<0.01)	0.46<0.01
After 3 months of treatment	0.84(<0.01)	0.76(<0.01)	0.87<0.01
After 6 months of treatment	0.81(<0.01)	0.88(<0.01)	0.89<0.01

**Table 5:** UWQOL v4.1-N scores based on the stage of the tumoura and type of treatment (Surgery vs Surgery + RT) b.

Item	Early stage		Advanced Stage		P value	Surgery		Surgery+RT		P value
	Mean (SD)	Mean Rank	Mean (SD)	Mean Rank		Mean (SD)	Mean Rank	Mean (SD)	Mean Rank	
Pain	94.85 (10.26)	126.21	62.50 (41.50)	83.53	<0.01	72.99 (38.36)	98.1	64.47 (40.70)	85.3	0.07
Appearance	80.15 (10.26)	132.79	48.14 (34.39)	82.01	<0.01	58.05 (32.90)	97.9	50.53 (34.20)	85.6	0.03
Activity	90.44 (12.33)	139.56	52.53 (36.62)	80.86	<0.01	63.79 (35.72)	97.8	55.79 (37.1)	85.6	0.09
Recreation	91.64 (12.21)	149.50	56.93	78.18	<0.01	70.11 (37.52)	99.7	60.26 (38.85)	83.9	0.03
Swallowing	93.82 (12.31)	140.97	49.26 (38.39)	80.14	<0.01	61.49 (37.99)	96.2	54.00 (39.93)	87.1	0.22
Chewing	89.71 (20.52)	147.59	40.54 (29.36)	78.61	<0.01	51.72 (32.75)	94.3	47.89 (34.91)	88.9	0.44
Speech	92.86 (11.98)	152.00	52.09 (37.20)	77.60	<0.01	65.06 (37.03)	96.4	57.37 (39.44)	86.9	0.19
Shoulder	74.12 (26.53)	109.51	55.61 (40.53)	87.36	<0.01	61.15 (37.12)	92.9	57.16 (40.6)	90.1	0.7
Taste	93.82 (12.31)	133.15	54.26 (40.53)	81.93	<0.01	64.37 (37.96)	93.4	59.16 (42.09)	89.7	0.62
Saliva	73.24 (39.67)	118.12	49.53 (38.39)	85.39	<0.01	57.36 (39.01)	95.5	50.84 (40.12)	87.7	0.30
Mood	89.71 (20.52)	136.13	49.93 (38.93)	81.25	<0.01	62.47 (39.0)	98.53	52.68 (39.12)	85.0	0.07
Anxiety	85.59 (28.73)	129.31	55.95 (38.86)	82.81	<0.01	66.21 (38.50)	98.7	57.16 (38.91)	84.9	0.03
Intimacy	94.71 (11.60)	134.29	54.73 (41.51)	81.67	<0.01	65.75 (39.32)	95.1	58.95 (42.12)	88.2	0.35
Fear of Recurrence	84.56 (20.42)	125.91	53.38 (38.98)	83.59	<0.01	63.79 (37.89)	98.5	55.0 (38.18)	85.01	0.07
Composite	82.39 (11.32)	144.50	61.18 (13.67)	79.32	<0.01	67.36 (14.62)	98.55	62.74 (18.07)	85.05	0.08

Man Whitney U test was used to compare the difference between the two groups. a. Base line evaluation. b. after 3 months of treatment

**Figure 1:**
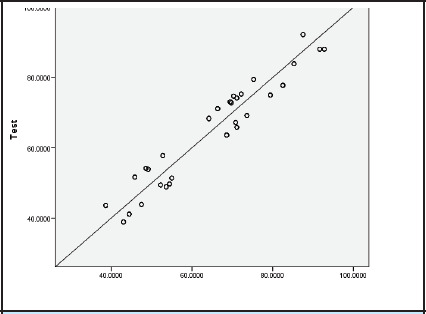
Test-retest Score

Test-retest reliability. Patients were asked to complete the same questionnaire 10 days after their initial evaluation. We got a strong correlation between the first and second evaluations with a correlation coefficient of 0.93 (N =203). (Fig 1)

## DISCUSSION

Quality of Life (QoL) is defined as the disparity between a patient's expectations and their current reality.^12^ It involves a multidimensional assessment of the impact of a disease and its treatment on the patient's overall well-being, encompassing physical, social, psychological, cognitive, and emotional domains.^13,14^ QoL is pivotal in guiding shared decision-making, especially when treatment options present similar survival rates but differing morbidity profiles. ^15^ QoL is often assessed using questionnaires developed in English-speaking, developed countries. These require rigorous translation and revalidation for use in other cultures.^16^ The UWQOL questionnaire, is a well- validated and concise head and neck cancer scale, ^9^ we successfully adapted for the Nepali population.

This study included 203 treatment-naive head and neck cancer patients, with a male-to-female ratio of 3.14, a distribution consistent with existing literature.^17,18^ Most patients were diagnosed with oral cancer, with the buccal mucosa and alveolar complex being the most commonly affected subsites, as observed in the South Asian subcontinent.^19,20^ A majority of patients presented with advanced-stage disease (Stages III and IV). Patients in the early stages had significantly better QoL scores across all domains compared to those in the late stages, a finding supported by previous studies demonstrating the greater adverse impact of advanced disease on QoL.^6, 21^

The Nepali version of the University of Washington Quality of Life questionnaire (UW-QOL v4.1-N) demonstrated strong psychometric properties, supporting its reliability and validity for assessing quality of life in head and neck cancer patients in Nepal. The translation process ensured face and content validity, as confirmed by a multidisciplinary panel of healthcare professionals and patients, thereby ensuring cultural relevance and conceptual equivalence. Internal consistency was high, with a Cronbach's alpha of 0.87, indicating that the items within the scale measure a cohesive underlying construct. Furthermore, item-total correlations for all domains exceeded the recommended threshold of 0.30 (range: 0.329-0.692), reinforcing the internal coherence of the tool. Construct validity was established through correlation with the EORTC QLQ-H&N35, a widely validated tool in head and neck oncology. Strong negative correlations were observed across all overlapping domains, consistent with the inverse scoring direction of the two tools (i.e., higher UW-QOL scores denote better quality of life, while higher EORTC scores often indicate greater symptom burden). Notably, the pain domain showed the strongest correlation (r = -0.77), underscoring the convergent validity of the pain-related constructs. Moreover, the UW-QOL v4.1-N composite score showed increasing correlation with global quality of life scores from baseline to 6 months post-treatment (r = 0.46 at baseline, rising to 0.89 at 6 months), highlighting the tool's responsiveness to clinical change over time. This responsiveness is critical for longitudinal QoL assessment in cancer patients undergoing treatment and recovery. The study also established discriminant validity, as the tool effectively distinguished between early- and late-stage tumors. Patients with early-stage disease consistently reported significantly higher scores across all domains and in the overall composite score, reflecting better quality of life outcomes compared to those with advanced-stage disease. This finding reinforces the utility of UW-QOL v4.1-N in stratifying patient-reported outcomes based on disease severity.

Finally, test-retest reliability was confirmed with good agreement between repeated administrations 10 days apart, an interval chosen to balance recall minimization with clinical stability. All domains demonstrated robust reproducibility.

The strengths of this study include its longitudinal design with a follow-up period of 6 months, as well as the successful translation and validation of one of the most widely used QOL questionnaires for head and neck cancer. Additionally, the study was conducted in a major cancer centers in Nepal, providing a robust patient sample. However, there were few limitations. Most patients in the study had oral cancer, which is consistent with the oral cavity being the most common subsite. Additionally, the predominance of patients presenting with advanced-stage tumours reflects a common challenge in the region.

## CONCLUSION

The Nepali version of UWQOLv4.1-N is a valid and reliable instrument to assess the QoL of HNC patients in Nepal.
